# A Meta-Analysis Evaluating the Colchicine Therapy in Patients With Coronary Artery Disease

**DOI:** 10.3389/fcvm.2021.740896

**Published:** 2021-12-09

**Authors:** Stefan Grajek, Michał Michalak, Tomasz Urbanowicz, Anna Olasińska-Wiśniewska

**Affiliations:** ^1^I Department of Cardiology, Poznan University of Medical Sciences, Poznań, Poland; ^2^Department of Computer Science and Statistics, Poznan University of Medical Sciences, Poznań, Poland; ^3^Department of Cardiac Surgery and Transplantology, Poznan University of Medical Sciences, Poznań, Poland

**Keywords:** colchicine, coronary artery disease, discontinuation, net clinical benefit, inflammation, cardiac outcomes

## Abstract

**Background:** Evidence from recent studies has shown the benefits of colchicine for patients with coronary artery disease. The aim was to assess the effect of colchicine treatment on cardiovascular events, with an estimation of the risk of discontinuation and net clinical benefit.

**Methods and Results:** Fourteen trials with a total of 13,186 patients were selected through a systematic search. Colchicine therapy significantly reduced the relative risk of primary endpoint by about 30% [RR 0.70 (95%CI:0.56–0.88)]. Compared with placebo, colchicine significantly reduced the risk of ischemia-driven revascularization [RR 0.57 (95%CI 0.41–0.80)], ischemia-driven revascularization and resuscitation [RR 0.50 (95%CI 0.34–0.73)], myocardial infarction [RR 0.73 (95%CI 0.57–0.95)], and stroke [RR 0.49 (95%CI 0.30–0.7)]. Patients treated with colchicine in comparison with placebo have a significant increase in the risk of treatment cessation (RR 1.60 95%CI 1.06–2.42). However, in the analysis which excluded studies without placebo, the relative risk of discontinuation was smaller (RR 1.34 95%CI 0.97–1.84) and in the three largest studies, the risk of discontinuation was lower and insignificant [RR 1.26 (95%CI 0.87–1.83)]. The net clinical benefit was 17.8/1,000 patients (*p* < 0.001).

**Conclusion:** In coronary artery disease, low-dose colchicine significantly reduces the risk of the primary composite endpoint by about 30%. The drug should be considered as part of the preventive treatment in patients with good tolerance.

## Introduction

Colchicine is an established treatment for gout, Behcet's Disease, and Familial Mediterranean Fever (1). Recent literature suggests that colchicine has cardiovascular benefits in patients with coronary artery disease (CAD), with a decrease in the risk of myocardial infarction and other cardiac outcomes by reducing inflammation (2–4). Colchicine at a dose of 0.5 mg once daily reduces inflammasome (NLRP3) activation and neutrophil degranulation (5, 6). Additionally, in patients with CAD with high leukocyte activation (> 7,500 WBC/mm^2^), endothelial function is significantly improved (7). Vaidya et al. (8) suggested that low-dose colchicine therapy in the post-acute coronary syndrome (ACS) of patients favorably modifies coronary plaque, independent of high-dose statin intensification therapy and substantial low-density lipoprotein reduction. The same group of researchers proved that colchicine inhibits neutrophil extracellular trap formation in ACS post percutaneous coronary intervention (PCI) (9). In addition, colchicine is a relatively safe and low-cost medication, which has been available for many years. Since 2019, three large clinical randomized studies have been published that proved a favorable effect of colchicine on cardiovascular events in patients with CAD (10–12). However, some meta-analyses did not confirm these results (13–15). These inconclusive observations were the source of the present systematic review and meta-analysis. The aim was to assess the effect of colchicine treatment on cardiovascular events. Additionally, to the best of our knowledge, this is the first estimation of the risk of discontinuation of colchicine therapy and net clinical benefit.

## Methods

The study was conducted following the preferred reporting items for meta-analysis (PRISMA) recommendations and was registered with the International Prospective Register of Systematic Reviews (PROSPERO).

### Search Strategy and Selection Criteria

We conducted a systematic search of studies in PubMed, Embase, The Cochrane Library, and Web of Science until February 11, 2021. The keywords used in the search process were as follows: (“colchicine”) AND (“coronary artery disease” OR “CAD” OR “coronary heart disease” OR “CHD” OR “acute coronary syndrome” OR “ACS” OR “myocardial infarction” OR “MI” OR “angina” OR “ischemic heart disease” OR “percutaneous coronary intervention” OR “PCI”).

**Figure 1:**
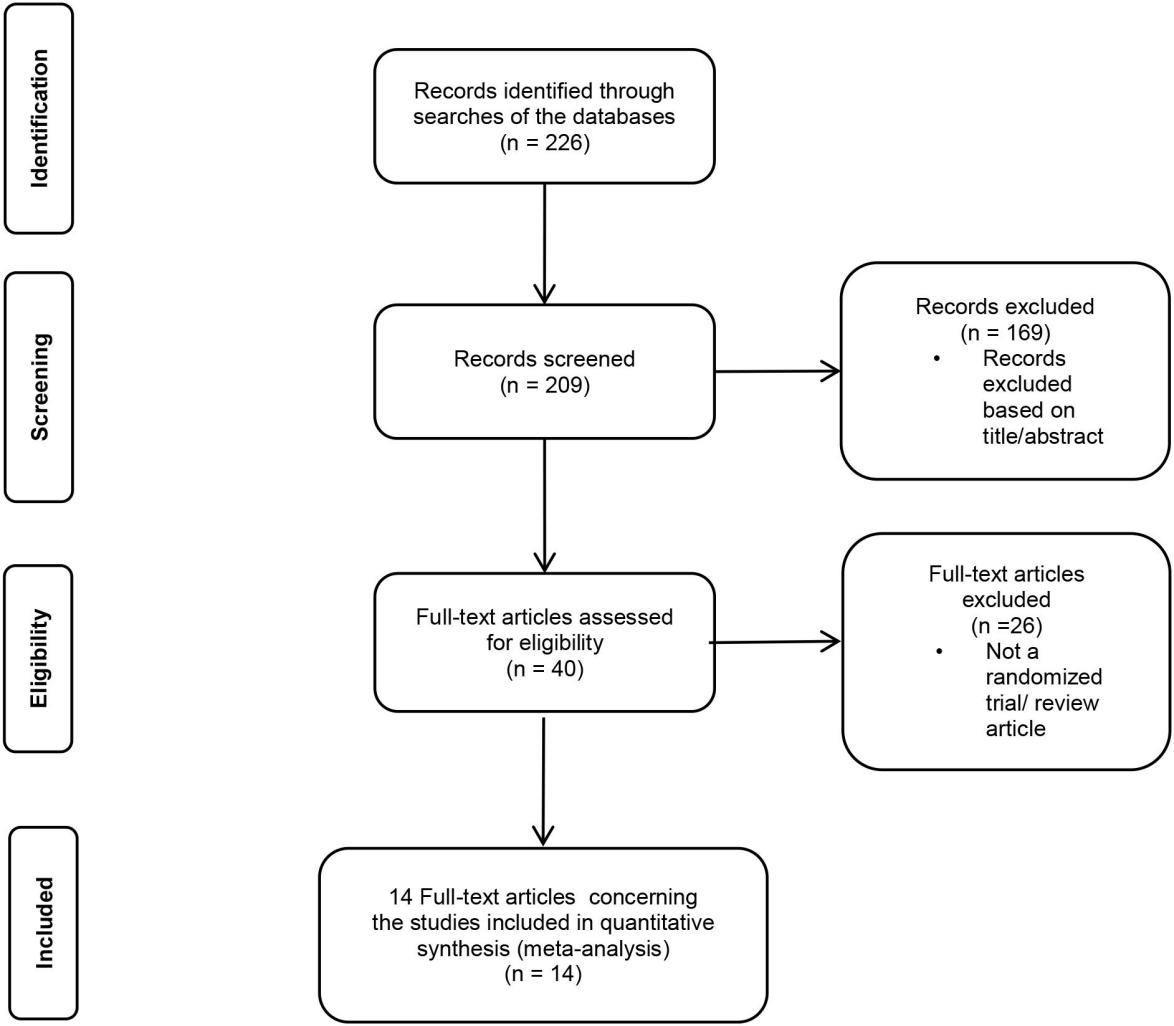
Preferred reporting items for meta-analysis (PRISMA) flow diagram of literature search.

The analysis included patients with CAD and ACS. Studies that fulfilled the following inclusion criteria were selected for the meta-analysis: 1. randomized controlled trial (RCT), comparing the effect of colchicine vs. placebo CAD or ACS patients, 2. reported study outcomes consistent with those focused in the present meta-analysis, 3. minimum follow-up period of 6 months. Registries, published abstracts, and meeting presentations were excluded. Finally, 14 studies were included in the analysis (Figure 1).

### Study Outcomes

Study outcomes were primary endpoints as defined in the particular study, other cardiac outcomes, and high-sensitive C-reactive protein (hs-CRP) (Table 1).

**Table 1:** Study outcomes.

	**Primary endpoint**	**Cardiovascular mortality**	**Ischemia driven revascularization**	**Ischemia driven revascularization + resuscitation**	**Myocardial infarction**	**Stroke**	**Death**	**Hs CRP**
Nidorf (11)	+	+	+		+	+	+	
Shah (16)	+				+		+	+
Tong (12)	+	+	+		+	+	+	
Tardif (10)	+	+	+	+	+	+	+	
Nidorf (17)	+	+	+	+	+	+	+	
Deftereos (18)		+					+	
Deftereos (19)		+	+			+	+	
Hennessy (20)			+		+			+
Akodad (21)					+			
Raju (22)						+		+
O'Keefe (23)							+	
Kajikawa (7)								+
Vaidya (8)								+
Nidorf (24)								+

*Hs CRP, high sensitive C-reactive protein*.

### Data Synthesis and Analysis

The methodological quality of randomized studies was assessed using the Cochrane Collaboration tool for assessing the risk of bias. For each clinical trial, bias was assessed qualitatively as low, unclear, or high (Supplementary Table 1). The assessment was made independently by two authors (SG and MM). To compare the results of colchicine vs. placebo patients, a meta-analysis using a random model was performed. As a measure of the effect, the Mantel-Haenszel relative risk (RR) with 95% confidence interval was used and in the case of hs-CRP analysis, the effect was measured as standardized mean difference (SMD). The data in the analyzed studies reported hs-CRP either as mean and SD or median and interquartile ranges (IQR). To enable the analysis for all those studies, we assumed the normal distribution of data and used the relationship between analyzed descriptive statistics: mean = median and SD = IQR/1.35. As a measure of heterogeneity, I2 statistics based on the Q-Cochran test were used. We calculated the net clinical benefit as the difference between the primary endpoint reduction and the increase of discontinuation ratio in colchicine vs. placebo patients. The result is expressed as a number of events per 1,000 patients.

Additionally, we performed a sensitivity analysis taking into account studies with a sample size greater than 100 in both arms and the follow-up was longer than 6 months. The calculations were performed using Review Manager (RevMan 5.3 Cochrane Community. Copenhagen: The Nordic Cochrane Center, The Cochrane Collaboration, 2014). A prospective protocol was uploaded to the PROSPERO online platform, with the registration number CRD42020218138.

## Results

A total of 226 studies were examined for eligibility, of which 14 papers were finally selected. A total of 13,186 patients who met the inclusion criteria (Figure 1) were included in the final analysis. A total number of 11,790 patients were included in the sensitivity analysis (Supplementary Material). Baseline patient characteristic for each study is summarized in Table 2. The mean age of the patients ranged from 56.3 to 68.7 years, with men accounting for 82.7% of the population. Ten trials used placebo and four were open-label type.

**Table 2:** Baseline patient characteristics.

**References**	**Type of comparator**	***N***	**Age**	**Male**	**Diagnosis**	**Previous Stroke**	**DM**	**HA**	**HL**	**Previous MI/ACS**	**Previous PCI**	**Previous CABG**	**HF**	**Tobacco smoking**
O'Keefe (23)	Colchicine Placebo	130 67	59 62	111 (85) 58 (87)	SCAD	-	16 (12) 8 (12)	-	-	-	-	34 (26) 17 (25)	-	-
Nidorf (24)	Colchicine Control	44 20	62 ± 10 59 ± 10	36 (81) 17 (85)	SCAD	-	-	-	-	-	-	-	-	-
Raju et al. (22)	Colchicine Placebo	40 40	57.2 (11.7) 57.2 (8.7)	34 (85) 37 (92.5)	UA 8 (20) UA 3 (7.5) MI 27 (67.5) MI 35 (87.5)	3 (7.5) 0 (0)	7 (17.5) 6 (15)	19 (47.5) 15 (37.5)	19 (47.5) 19 (47.5)	8 (20) 6 (15)	-	-	1(2.5) 0 (0)	18 (45) 17 (42.5)
Nidorf et al. (17) LODoCO	Colchicine Control	282 250	66 ± 9.6 67 ± 9.2	251 (89) 222 (89)	-	-	92 (33) 69 (28)	-	-	MI or UA 64 (23) 61 (24)	169 (60) 138 (55)	62 (22) 39 (16)	-	10 (4) 14 (6)
Deftereos et al. (19)	Colchicine Placebo	100 96	63.7 ± 6.9 63.5 ± 7.2	63 (63) 65 (68)	SCAD 72 (72) SCAD 63 (66) ACS 28 (28) ACS 33 (34)	-	100 (100) 96 (100)	48 (48) 47 (49)	-	-	-	-	-	36 (36) 38 (40)
Deftereos et al. (18)	Colchicine Placebo	140 139	66.9 ± 5.8 66.4 ± 5.7	94 (67) 93 (67)	Stable HF	-	23 (16) 25 (18)	48 (34) 53 (38)	46 (33) 45 (32)	-	-	-	all	-
Vaidya (8)	Colchicine Control	40 40	56.3 ± 8.9 58.4 ± 14.2	32 (80) 30 (75)	STEMI 3 (7.5) 6 (15) NSTEMI 14 (35) 16 (40) UA 23 (57.5) 16 (40)	-	9 (22.5) 16 (40)	20 (50) 23 (57.5)	34 (85) 34 (85)	19 (47.5) 22 (55)	28 (70) 23 (57.5)	-	-	20 (50) 21 (52.5)
Akodad et al. (21) COLIN	Colchicine Control	23 21	60.1 ± 13.1 59.7 ± 11.4	19 (82.5) 16 (76.2)	STEMI	-	3 (13.0) 3 (14.3)	9 (39.1) 10 (47.6)	8 (34.8) 8 (38.1)	-	1 (4.3) 1 (4.8)	0 (0) 1 (4.8)	9 (39.1) 5 (23.8)	17 (73.9) 14 (66.7)
Tardif et al. (10) COLCOT	Colchicine Placebo	2366 2379	60.6 ± 10.7 60.5 ± 10.6	1894 (80) 1942 (81.6)	CAD within 30 days after MI	55 (2.3) 67 (2.8)	462 (19.5) 497 (20.9)	1185 (50.1) 1236 (52)	-	370 (15.6) 397 (16.7)	392 (16.6) 406 (17.1)	69 (2.9) 81 (3.4)	48 (2) 42 (1.8)	708 (29.9) 708 (29.8)
Hennessy et al. (20)	Colchicine Placebo	119 118	61 ± 13.6 61 ± 12.5	89 (75) 93 (79)	Acute MI, including STEMI 63 (53) 71 (60)	-	27 (23) 25 (21)	64 (54) 48 (41)	-	18 (15) 18 (15)	Prior revascularisation 13 (11) 14 (12)		-	-
Kajikawa (7)	Colchicine Placebo (data for whole group)	28	68 ± 7	27 (96.4)		1 (3.6)	10 (35.7)	25 (89.3)	-	16 (57.1)	20 (71.4)	1 (3.6)		Current 8 (28.6) Former 27 (96.4)
Shah et al. (16) COLCHICINE-PCI	Colchicine Placebo	206 194	65.9 + 9.9 66.6 ± 10.2	193 (93.7) 181 (93.3)	ACS 103 (50.0) ACS 95 (49.0)	-	114 (55.3) 117 (60.3)	192 (93.2) 175 (90.2)	182 (88.3) 173 (89.2)	51 (24.8) 52 (26.8)	-	-	-	43 (20.9) 46 (23.7)
Nidorf et al. (11) LoDoCo 2	Colchicine Placebo	2762 2760	65.8 ± 8.4 65.9 ± 8.7	2305 (83.5) 2371 (85.9)	CAD	-	492 (17.8) 515 (18.7)	1421 (51.4) 1387 (50.3)	-	2323 (84.1) 2335 (84.6)	2100 (76.0) 2077 (75.3)	319 (11.5) 391 (14.2)	-	318 (11.5) 330 (12.0)
Tong et al. (12) COPS	Colchicine Placebo	396 399	59.7 ± 10.2 60.0 ± 10.4	322 (81) 310 (78)	STEMI 182 (48) STEMI208 (53) NSTEMI 183 (48) NSTEMI 174 (44) UA 15 (4) UA 11 (3)	5 (1) 11 (3)	75 (19) 76 (19)	201 (51) 199 (50)	180 (46) 185 (46)	59 (15) 59 (15)	51 (13) 50 (13)	15 (4) 19 (5)	-	128 (32) 149 (37)

*ACS, acute coronary syndrome; CABG, coronary artery bypass grafting; CAD, coronary artery disease; DM, diabetes mellitus; HA, arterial hypertension; HF, heart failure; HL, hyperlipidaemia; MI, myocardial infarction; NSTEMI, non ST elevation myocardial infarction; PCI, percutaneous intervention; SCAD, stable coronary artery disease; STEMI, ST elevation myocardial infarction, UA, unstable angina*.

### Efficacy Endpoints

**Figure 2:**
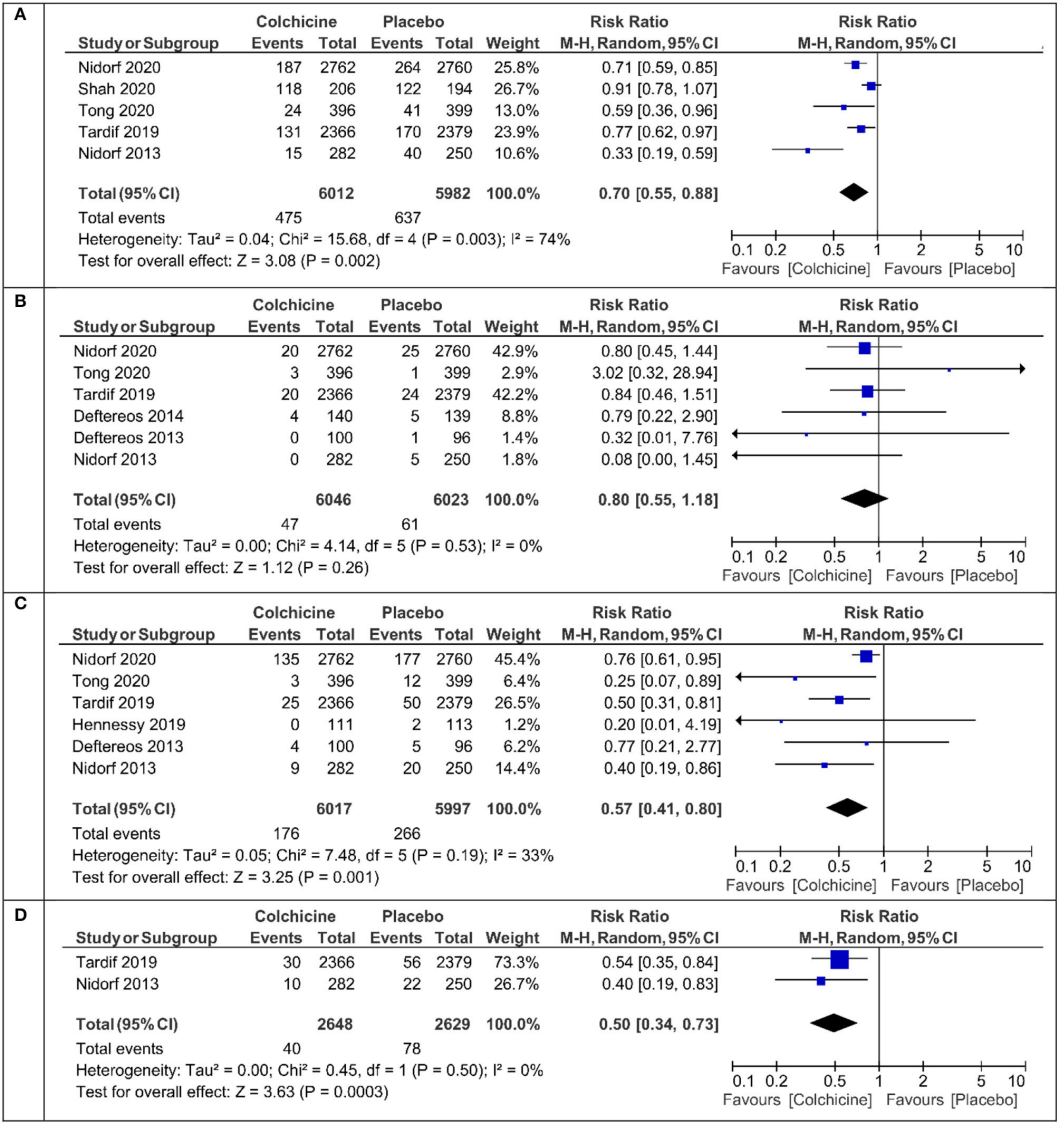
Meta-analysis results for the primary endpoint, cardiovascular death, ischemia driven revascularization, and ischemia driven revascularization + resuscitation. **(A)** Primary endpoint, **(B)** cardiovascular mortality, **(C)** ischemia driven revascularization, **(D)** ischemia driven revascularization + resuscitation.

**Figure 3:**
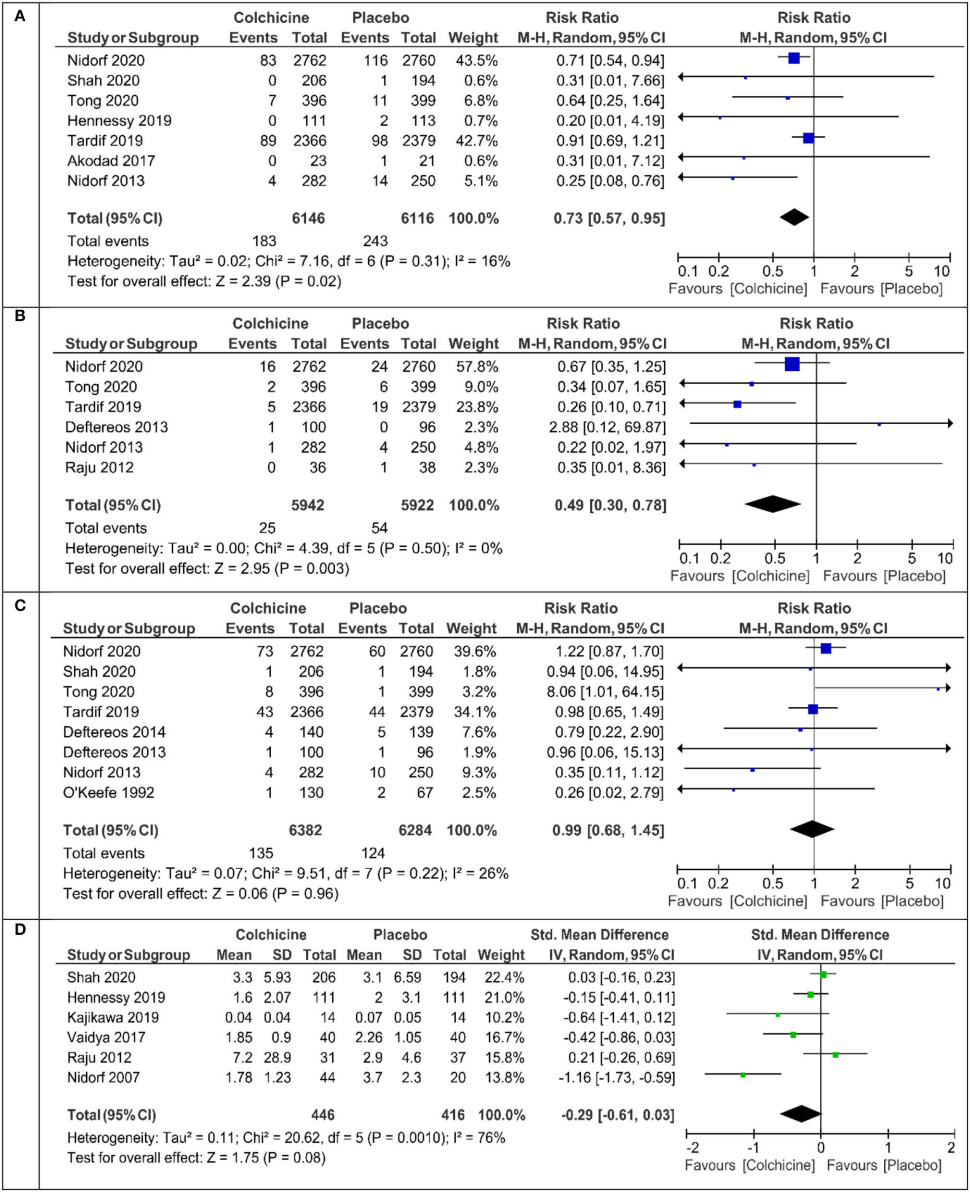
Meta-analysis results for myocardial infarction, stroke, all-cause death, and high-sensitive C-reactive protein (hs-CRP). **(A)** Myocardial infarction, **(B)** stroke, **(C)** death, **(D)** hs-CRP.

**Figure 4:**
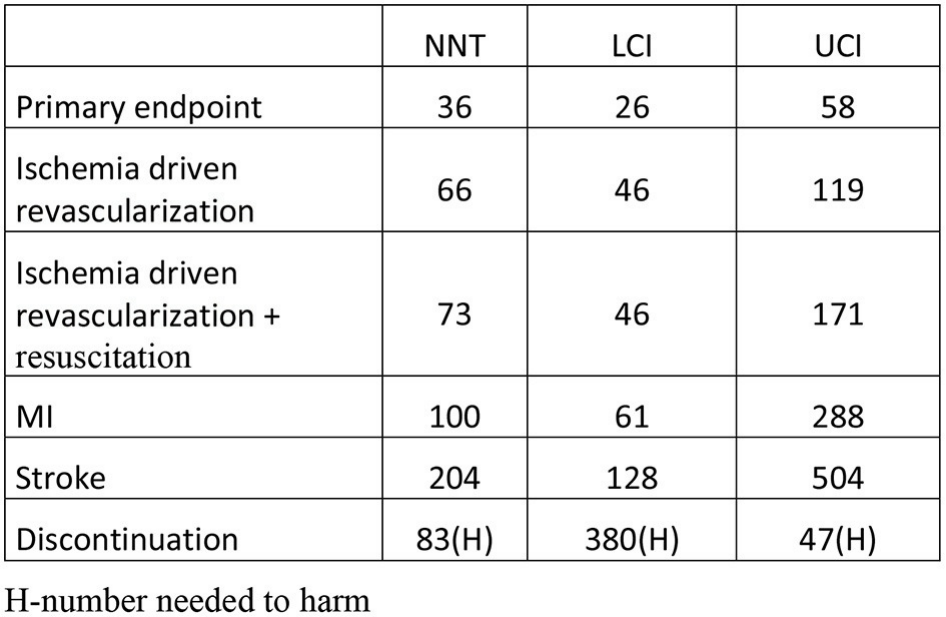
The number needed to treat (NNT) for analyzed endpoints.

The primary endpoint defined as cardiovascular death or myocardial infarction or ischemic stroke or ischemia driven revascularization or PCI-related myocardial injury was calculated in five studies (Table 1). Colchicine therapy significantly reduced the relative risk coefficient of the primary endpoint by about 30% [RR 0.70 (95% CI:0.56–0.88), Figure 2A]. In the sensitivity analysis which included four studies (Supplementary Figure 2A), the risk of the primary endpoint was similar [RR 0.64 (95% CI 0.50–0.82)]. Compared with placebo, colchicine significantly reduced the risk of ischemia driven revascularization [RR 0.57 (95% CI 0.41–0.80), Figure 2C], ischemia driven revascularization and resuscitation [RR 0.50 (95% CI 0.34–0.73), Figure 2D], myocardial infarction [RR 0.73 (95% CI 0.57–0.95), Figure 3A], and stroke [RR 0.49 (95% CI 0.30–0.7), Figure 3B]. In the sensitivity analysis, the results were consistent and always significant (Supplementary Figures 2, 3). Colchicine did not significantly reduce the risk of cardiovascular death and overall death (Figures 2B, 3C). Colchicine therapy was associated with a significant reduction in hs-CRP level (Figure 3D). Figure 4 presents the Number Need to Treat (NNT) for the significantly reduced primary endpoint and other cardiovascular events.

### Safety Endpoints

**Figure 5:**
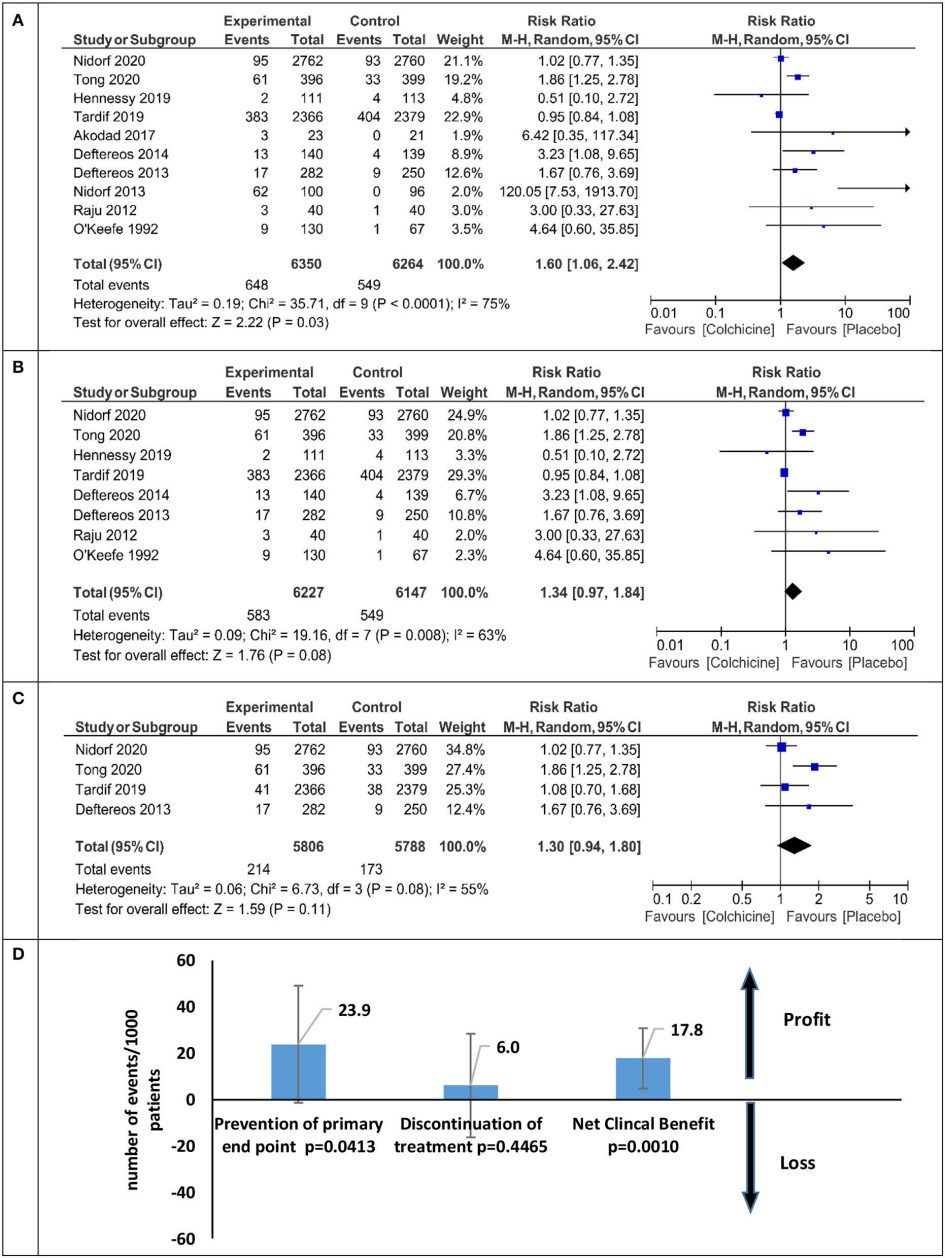
Meta-analysis for the therapy discontinuation and net clinical benefit for all analyzed studies and sensitivity analysis. **(A)** Colchicine therapy discontinuation-all studies, **(B)** colchicine therapy discontinuation without PROBE studies, **(C)** colchicine therapy discontinuation sensitivity analysis, **(D)** net clinical benefit sensitivity analysis.

The overall relative risk (RR) of discontinuation with colchicine therapy is presented in Figure 5A. Patients treated with colchicine in comparison with placebo have a significant increase in the risk of treatment cessation (RR 1.60 95% CI 1.06–2.42), but the analysis of excluded studies without placebo (PROBE studies) had a lesser relative risk of discontinuation (RR 1.34 95% CI 0.97–1.84) (Figure 5B). After the inclusion of only the three largest studies, the RR of discontinuation was the lowest and insignificant [RR 1.26 (95% CI 0.87–1.83), Figure 5C]. The net clinical benefit calculated as the difference between primary endpoint reduction and discontinuation ratio in colchicine vs. placebo patients was 17.8/1,000 patients (*p* < 0.001) and is presented in Figure 5D.

## Discussion

The results of our meta-analysis prove that treatment with colchicine at a dose 0.5 mg once daily in patients with CAD significantly reduces the primary endpoint composed of cardiovascular mortality, myocardial infarction, stroke, and urgent ischemia-driven revascularization by 30% (relative risk). The estimated NNT is 36 (26–58) on average. This result does not confirm the negative results of other meta-analyses, which have shown a favorable reduction of ischemic events, although the differences were not significant (13–15). Additionally, we proved that colchicine treatment significantly reduces the relative risk of myocardial infarction (27%), ischemia-driven revascularization (43%), and stroke (51%). Our results are consistent with the two most recent meta-analyses (25, 26). The reasons for this discrepancy should be seen in the number of patients included in the assessment. Among all cited meta-analyses, ours contains the largest number of 13,186 (7, 8, 10–12, 16–24). After excluding small observational clinical trials, 11,790 patients were included in the sensitivity meta-analysis and the results were almost identical to those in the main meta-analysis (Supplementary Material). This confirms the reliability and robustness of the obtained results.

Like all other investigators, we did not find a significant effect of the drug on cardiovascular and total mortality. In the cited studies, the beneficial effect of colchicine treatment was observed in both chronic (CCS) and acute (ACS) coronary syndromes. In the latter, the therapeutic efficacy of colchicine was greater the sooner the treatment was introduced after a myocardial infarction (the best period up to 3 days) (27). Almost all studies showed a decrease in serum hs-CRP, but the presentation of results by individual authors varied. We managed to extract the result in the form of the mean +/- SD from six studies. In patients after treatment with colchicine (*n* = 446), the mean value of hs-CRP was 1.78 mg% compared with 3.7 mg% in the placebo group (*n* = 416) (Figure 3D). In patients with ACS, elevated levels of hs - CRP, interleukin-1, and interleukin-6 are indicators of the risk of cardiovascular complications (28–31). Treatment with colchicine in comparison with placebo leads to a greater reduction in the volume of atherosclerotic plaque, and changes in its volume also correlate with changes in serum hs-CRP levels (8). Therefore, in patients with CAD, the combination of colchicine with aggressive statin therapy seems to be justified and desired (28, 30).

Generally, colchicine is well tolerated. However, in many studies, several side effects were observed. Compared with placebo, the occurrence of diarrhea (17.9 vs. 13.1%) and gastrointestinal disorders (17.6 vs 13.1%) was significantly more frequent. There were no significant increase in hepatic, muscle, infectious, hematological complications, sensory disturbances, serious side effects, and deaths (2, 32). The dose of colchicine used in cardiovascular diseases is 0.5 mg once daily and is lower than in the treatment of rheumatoid diseases.

In patients with cardiovascular diseases, simple gastrointestinal side effects are most common, leading them however to discontinue treatment. We found data on patients who discontinued the drug because of side effects in 10 studies. Colchicine was discontinued by 10.2% (648/6,350) of the patients and by 8.7% (549/6,264) of the placebo/control group. Compared with placebo, the relative risk of colchicine withdrawal was 60% (RR 1.60 95% CI 1.06–2.42) (Figure 5A). After excluding the non-placebo trials (21, 26, 33), the risk of withdrawal was reduced by half (RR 1.34 95% CI.97–1.84) (Figure 5B). We calculated the Net Clinical endpoint (colchicine vs. placebo) as the difference between the reduction of the composite endpoint (benefit) and the increase in the number of patients discontinuing treatment due to side effects (harm). However, in individual studies, the follow-up time varied significantly from 1 to 28 months, not all studies used a placebo (PROBE study) (17, 21, 33), and the sample size ranged from 44 to 5,522 patients. In the three largest studies with a composite endpoint, a placebo was used, the follow-up was over 6 months, and the number of subjects was over 100 in each arm (10–12), while the risk of discontinuation of colchicine treatment was reduced to 26% (Figure 5C).

Taking into account the risk of discontinuation of colchicine treatment, the estimated potential benefit from the continued use of the drug covers 18/1,000 patients and this ratio is statistically significant (*p* = 0.001, Figure 5D). From a clinical point of view, the values of NNT 36 (26–58) for the primary endpoint and NNH 83 (380–47) for the occurrence of side effects causing discontinuation are interesting and acceptable. In all studies, these favorable results were seen in patients treated with statins and antiplatelet drugs. Colchicine is a cheap drug, and if added to the standard treatment of patients with ischemic heart disease, it can significantly enhance the power of preventive therapy at a low cost. Although colchicine does not reduce the risk of cardiovascular mortality in patients with ACS and CCS, colchicine should be considered to reduce cardiovascular morbidity in patients who tolerate the drug.

### Study Limitations

Some limitations of this analysis should be addressed. First, the results of original studies are limited by the heterogeneity across the trials and the possibility of publication bias (acute vs. chronic coronary syndromes, gender, various age groups, various number of endpoints in the studies). However, we tried to minimize the influence of these limitations, including the application of the random-effects model and sensitivity analysis, incorporating to the analysis only the studies with both arms where the number of patients was above 100. Secondly, the definition of primary endpoints across the trials differed. That is why all components of the primary endpoint were analyzed separately. Such definition of primary endpoints also influenced the robustness of net clinical benefit. Finally, the hs-CRP results were presented in different descriptive statistics, and its analysis required some transformations.

## Conclusion

In CAD, low-dose colchicine (0.5 mg once daily) significantly reduces the risk of the primary composite endpoint by about 30%. The drug should be considered as part of the preventive treatment in patients with good tolerance. When added to standard therapy, it significantly reduces cardiovascular morbidity.

## Data Availability Statement

The raw data supporting the conclusions of this article will be made available by the authors, without undue reservation.

## Author Contributions

SG: conceptualization, methodology, analysis, original draft preparation, and supervision. MM: software, methodology, and draft editing. TU: analysis and draft editing. AO-W: analysis, methodology, and draft editing. All authors contributed to the article and approved the submitted version.

## Conflict of Interest

The authors declare that the research was conducted in the absence of any commercial or financial relationships that could be construed as a potential conflict of interest.

## Publisher's Note

All claims expressed in this article are solely those of the authors and do not necessarily represent those of their affiliated organizations, or those of the publisher, the editors and the reviewers. Any product that may be evaluated in this article, or claim that may be made by its manufacturer, is not guaranteed or endorsed by the publisher.
