# Supplementary material for: A Meta-Analysis Evaluating the Colchicine Therapy in Patients With Coronary Artery Disease
Source: Front Cardiovasc Med. 2021 Dec 9;8:740896. doi: 10.3389/fcvm.2021.740896 (PMC8696075; doi:10.3389/fcvm.2021.740896)
Supplement: Supplementary file 1 [file Data_Sheet_1.docx]

Figure S1. The Risk of bias of individual studies by Cochrane risk assessment tool.

| Study | Random sequence generation (Selection bias) | Allocation concealment *(Selection bias*) | Blinding of participants and personnel *(Performance bias)* | Blinding of outcome assessment personnel *(Detection bias)* | Incomplete outcome data *(Attrition bias)* | Selective reporting *(Reporting bias)* | Other sources of bias |
| --- | --- | --- | --- | --- | --- | --- | --- |
| Nidorf 2020 | 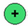 | 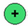 | 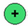 | 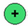 | 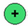 | 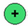 | 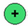 |
| Shah 2020 | 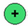 | 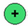 | 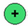 | 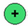 | 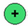 | 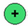 | 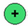 |
| Tong 2020 | 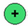 | 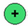 | 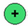 | 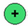 | 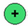 | 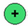 | 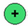 |
| Hennessy 2019 | 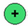 | 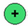 | 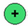 | 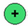 | 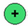 | 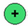 | 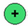 |
| Kajikawa 2019 | 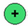 | 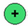 | 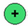 | 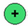 | 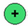 | 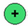 | 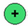 |
| Tardif 2019 | 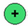 | 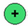 | 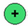 | 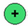 | 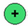 | 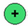 | 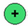 |
| Akodad 2017 | 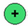 | 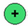 | 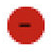 | 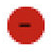 | 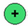 | 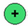 | 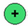 |
| Vaidya 2017 | 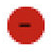 | 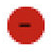 | 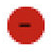 | 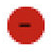 | **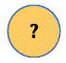** | 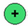 | 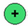 |
| Deftereos 2014 | 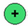 | 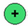 | 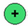 | 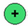 | 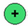 | 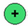 | 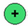 |
| Deftereos 2013 | 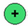 | **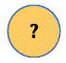** | 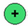 | 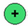 | 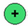 | 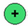 | 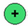 |
| Nidorf 2013 | 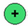 | 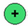 | 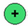 | 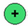 | 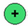 | 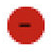 | 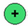 |
| Raju 2012 | 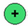 | 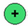 | 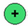 | 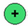 | 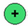 | 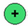 | 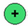 |
| Nidorf 2007 | **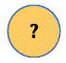** | **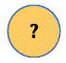** | **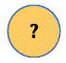** | **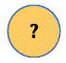** | 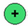 | 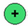 | 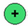 |
| O'Keefe 1992 | 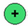 | 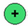 | 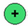 | 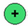 | 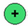 | 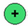 | 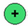 |

| 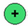 | **low risk of bias** | **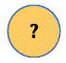** | **unclear risk of bias** |  | **high risk of bias** |
| --- | --- | --- | --- | --- | --- |

Figure S2. Meta-analysis results (sensitivity analysis) for primary endpoint, cardiovascular death, ischemia driven revascularization and ischemia driven revascularization + resuscitation.

| A |  |
| --- | --- |
| B |  |
| C |  |

A-Primary endpoint, B-Cardiovascular death, C-Ischemia driven revascularization.

Figure S3. Meta-analysis results (sensitivity analysis) for for myocardial infarction, stroke, all-cause death.

| A |  |
| --- | --- |
| B |  |
| C |  |

A-myocardial infarction, B-stroke, C-all-cause death
